# Correction: Within-patient mutation frequencies reveal fitness costs of CpG dinucleotides and drastic amino acid changes in HIV

**DOI:** 10.1371/journal.pgen.1007855

**Published:** 2018-12-10

**Authors:** Kristof Theys, Alison F. Feder, Maoz Gelbart, Marion Hartl, Adi Stern, Pleuni S. Pennings

The following information is missing from the Funding section: This study was supported by the Binational US-Israel Foundation grant 2016555 to AS.
