# Supplementary figures and images for: Within-patient mutation frequencies reveal fitness costs of CpG dinucleotides and drastic amino acid changes in HIV
Source: PLoS Genet. 2018 Jun 28;14(6):e1007420. doi: 10.1371/journal.pgen.1007420 (PMC6023119; doi:10.1371/journal.pgen.1007420)

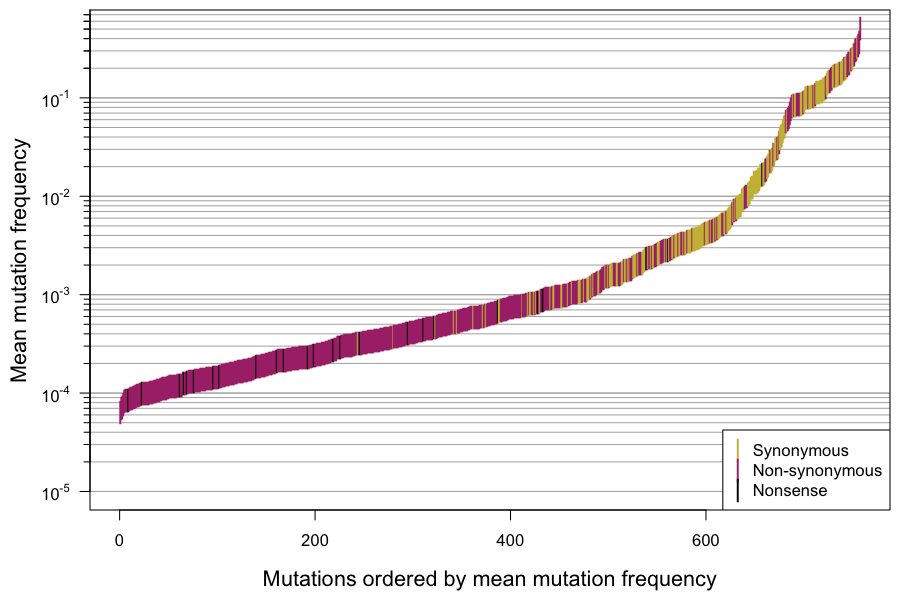

Supplement: S1 Fig — Mutation frequency for 758 pol sites from the Zanini dataset [45], ordered by mutation frequency. (PNG) [file pgen.1007420.s001.png]

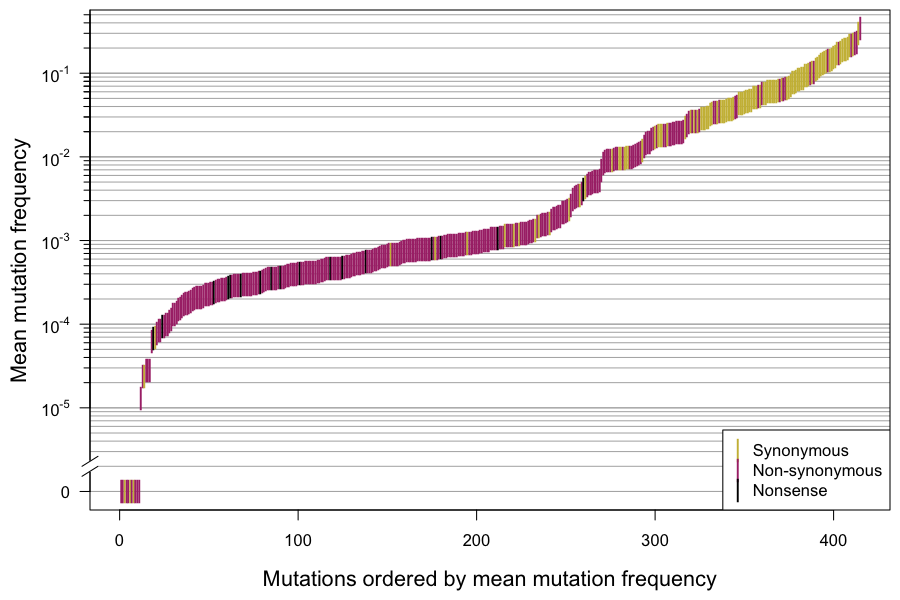

Supplement: S2 Fig — Mutation frequency for 415 reverse transcriptase sites from the Lehman dataset [46], ordered by mutation frequency. (PNG) [file pgen.1007420.s002.png]

**a : syn**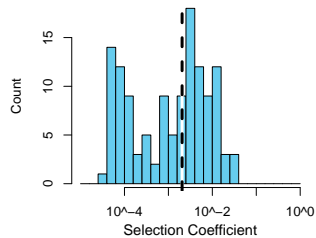**t : syn**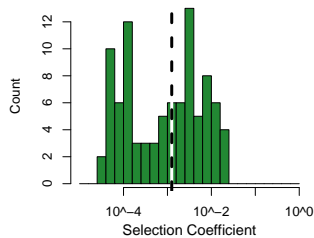**a : nonsyn**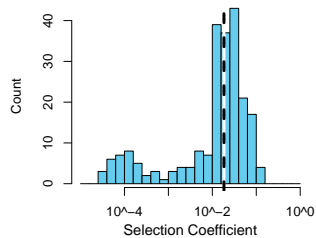**t : nonsyn**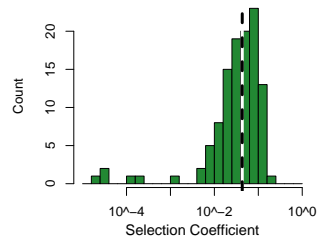**c : syn**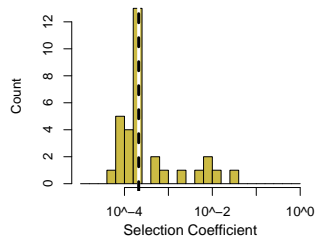**g : syn**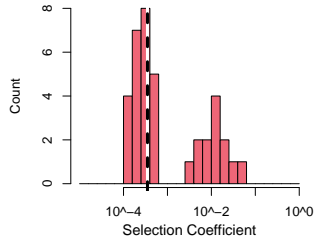**c : nonsyn**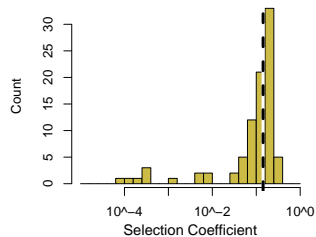**g : nonsyn**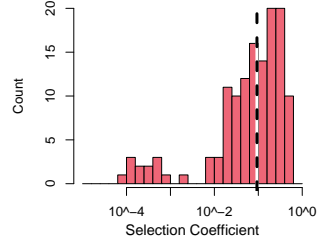

Supplement: S3 Fig — Distribution of fitness costs for non-synonymous and synonymous mutations for the Zanini dataset [45]. Nonsense mutations are included in the non-synonymous mutations. Note that the scale of the y-axis differs between the graphs. (PDF) [file pgen.1007420.s003.pdf]

**a : syn**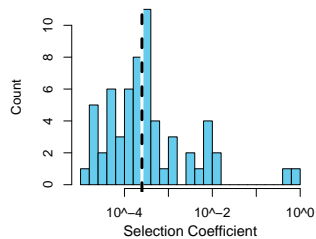**t : syn**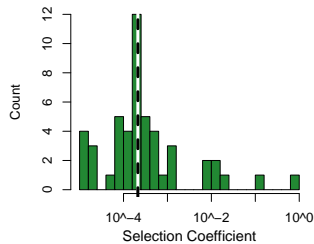**a : nonsyn**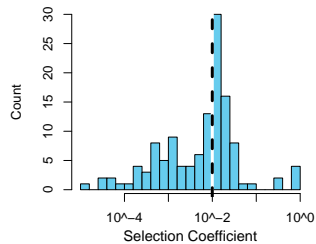**t : nonsyn**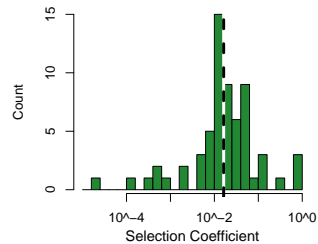**c : syn**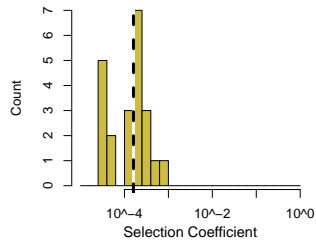**g : syn**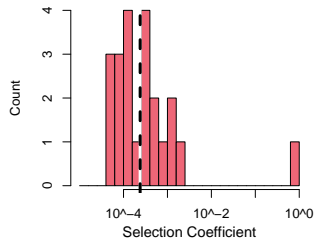**c : nonsyn**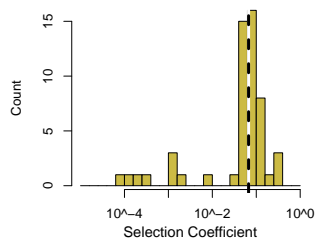**g : nonsyn**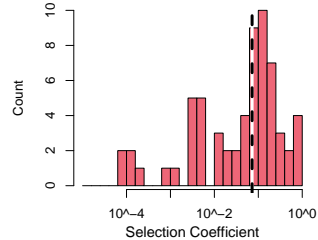

Supplement: S4 Fig — Distribution of fitness costs for non-synonymous and synonymous reverse transcriptase mutations from the Lehman dataset [46]. Nonsense mutations are included in the non-synonymous mutation category. Note that the scale of the y-axis differs between the graphs. (PDF) [file pgen.1007420.s004.pdf]

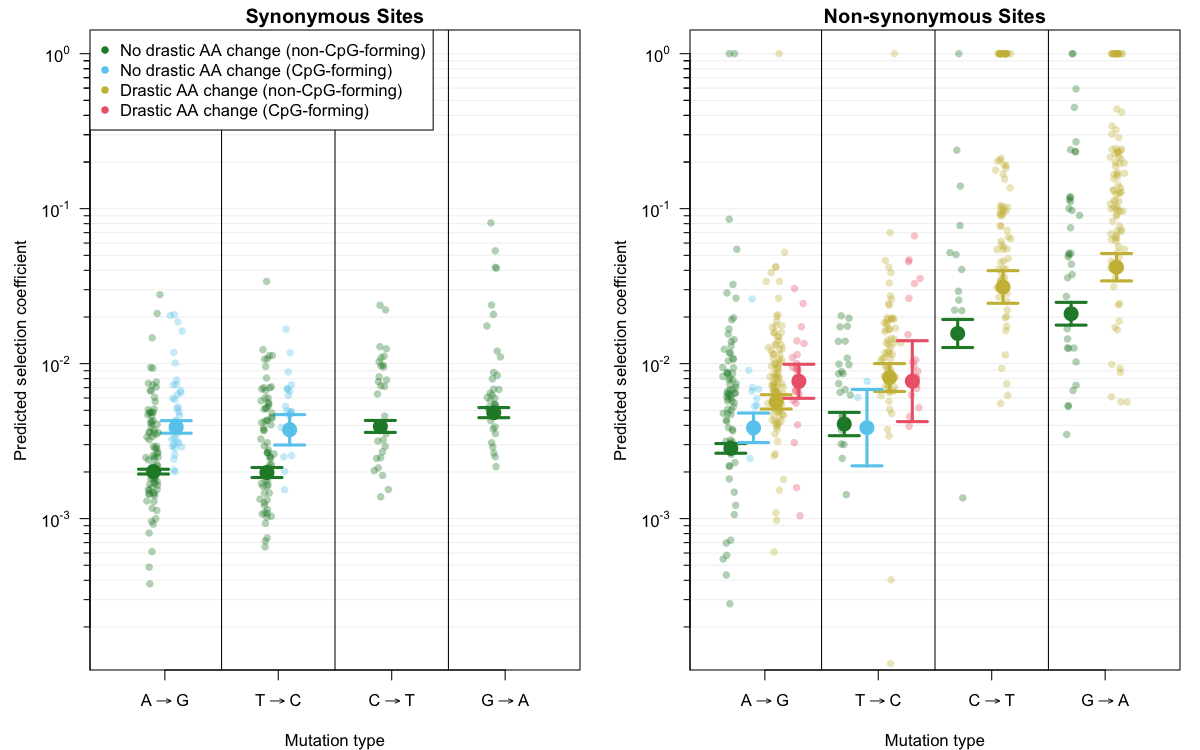

Supplement: S5 Fig — Selection coefficients for transitions at every nucleotide site in the pol sequence show that CpG-forming mutations are more costly than non-CpG-forming mutations and that mutations that involve a drastic amino acid change are more costly than mutations that do not. Selection coefficients were estimated using a generalized linear model and sequence data from 160 HIV-infected patients. Shown are predicted selection coefficients for synonymous (left) and non-synonymous (right) mutations that do not involve a drastic amino acid change and either create CpG sites (blue) or do not (green). For non-synonymous mutations, predictions are also shown for mutations that do involve drastic amino acid changes and either create CpG sites (light red) or do not (yellow). (PNG) [file pgen.1007420.s005.png]

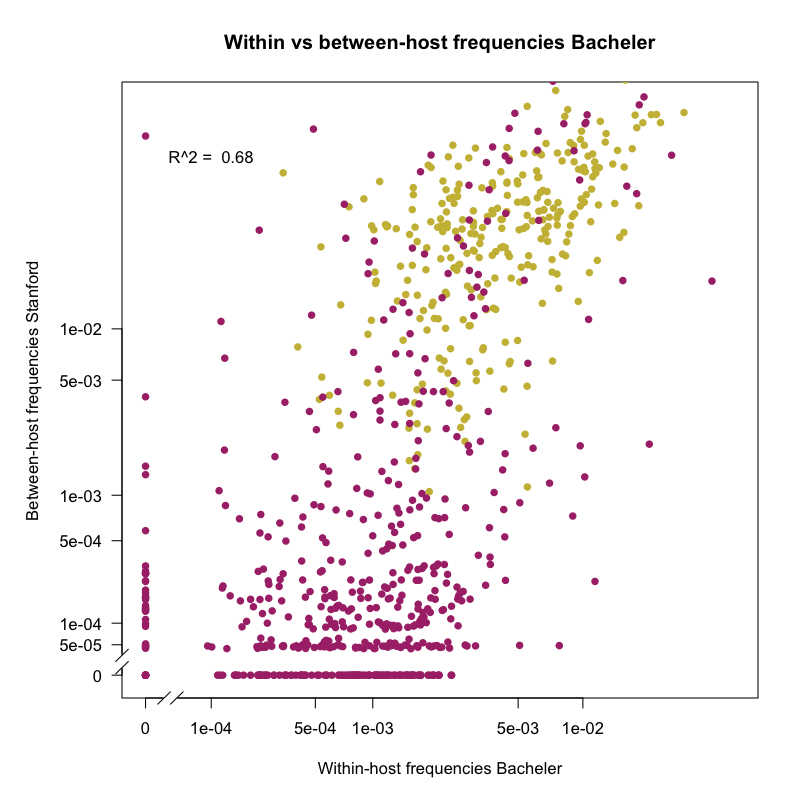

Supplement: S6 Fig — A correlation (Spearman’s rank correlation coefficient ρ = 0.68) exists between average pol mutation frequencies at the within-patient level (in the 160 patients analyzed in this study) and mutant frequencies in the global subtype B epidemic (23,742 protease and 22,785 reverse transcriptase consensus sequences from the HIVdb [47]). Values shown on a log scale. Non-synonymous mutations are shown in dark pink, synonymous mutations in yellow. (PNG) [file pgen.1007420.s006.png]

## Mutation rate estimates used

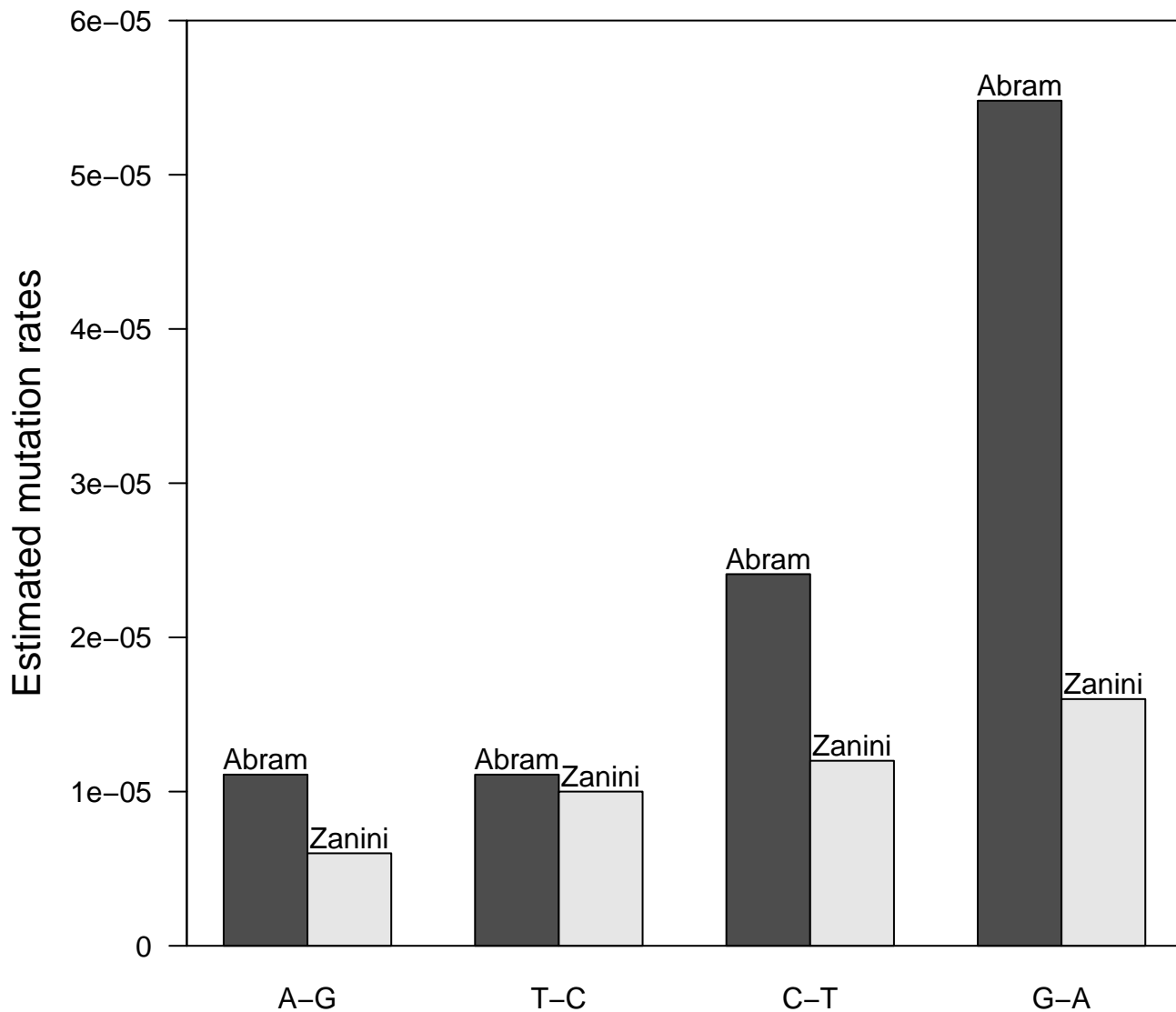

Supplement: S7 Fig — (PDF) [file pgen.1007420.s007.pdf]
