# Supplementary material for: Within-patient mutation frequencies reveal fitness costs of CpG dinucleotides and drastic amino acid changes in HIV
Source: PLoS Genet. 2018 Jun 28;14(6):e1007420. doi: 10.1371/journal.pgen.1007420 (PMC6023119; doi:10.1371/journal.pgen.1007420)
Supplement: S1 Table — All sites were grouped in 9 groups, then the 5% highest selection coefficients were recorded in each group. (PDF) [file pgen.1007420.s008.pdf]

	WT	MUT	num	HXB2	WTAA	MUTAA	bigAAChange	CpG	EstSelCoeff
49	g	a	49	2301	G	R	1	0	1.00
67	c	t	67	2319	L	L	0	0	0.02
79	g	a	79	2331	G	R	1	0	1.00
88	g	a	88	2340	D	N	1	0	1.00
99	a	g	99	2351	L	L	0	0	0.03
100	g	a	100	2352	E	K	1	0	1.00
112	t	c	112	2364	L	L	0	0	0.01
118	g	a	118	2370	G	R	1	0	1.00
124	t	c	124	2376	W	R	1	1	0.07
131	c	t	131	2383	P	L	1	0	1.00
141	a	g	141	2393	I	M	0	0	1.00
186	a	g	186	2438	I	M	0	0	0.09
202	g	a	202	2454	G	R	1	0	1.00
207	t	c	207	2459	H	H	0	0	0.03
218	g	a	218	2470	G	D	1	0	1.00
232	g	a	232	2484	G	R	1	0	1.00
235	c	t	235	2487	P	S	1	0	1.00
236	c	t	236	2488	P	L	1	0	1.00
270	g	a	270	2522	L	L	0	0	0.05
272	c	t	272	2524	T	I	1	0	1.00
278	t	c	278	2530	I	T	1	0	0.04
280	g	a	280	2532	G	S	1	0	1.00
287	c	t	287	2539	T	I	1	0	1.00
291	a	g	291	2543	L	L	0	0	0.02
293	a	g	293	2545	N	S	0	0	0.05
