# Supplementary material for: Within-patient mutation frequencies reveal fitness costs of CpG dinucleotides and drastic amino acid changes in HIV
Source: PLoS Genet. 2018 Jun 28;14(6):e1007420. doi: 10.1371/journal.pgen.1007420 (PMC6023119; doi:10.1371/journal.pgen.1007420)
Supplement: S2 Table — All sites were grouped in 9 groups, then the 5% highest selection coefficients were recorded in each group. (PDF) [file pgen.1007420.s009.pdf]

	WT	MUT	num	HXB2	WTAA	MUTAA	bigAAChange	CpG	EstSelCoeff
301	a	g	4	2554	I	V	0	1	0.03
324	a	g	27	2577	P	P	0	1	0.02
337	c	t	40	2590	P	S	1	0	1.00
340	g	a	43	2593	G	R	1	0	1.00
344	t	c	47	2597	M	T	1	1	0.05
349	g	a	52	2602	G	S	1	0	1.00
355	a	g	58	2608	K	E	1	0	1.00
371	c	t	74	2624	P	L	1	0	1.00
377	c	t	80	2630	T	I	1	0	1.00
390	a	g	93	2643	I	M	0	0	1.00
409	t	c	112	2662	C	R	1	1	0.05
415	g	a	118	2668	E	K	1	0	1.00
438	t	c	141	2691	I	I	0	0	0.01
440	c	t	143	2693	S	L	1	0	1.00
442	a	g	145	2695	K	E	1	0	0.04
464	a	g	167	2717	Y	C	1	0	0.04
475	g	a	178	2728	V	I	0	0	1.00
485	t	c	188	2738	I	T	1	0	0.05
486	a	g	189	2739	I	M	0	0	0.03
518	t	c	221	2771	L	S	1	0	0.07
530	g	a	233	2783	R	K	0	0	1.00
535	c	t	238	2788	L	F	0	0	1.00
583	c	t	286	2836	H	Y	1	0	1.00
587	c	t	290	2840	P	L	1	0	1.00
592	g	a	295	2845	G	R	1	0	1.00
603	g	a	306	2856	K	K	0	0	0.08
607	a	g	310	2860	K	E	1	0	0.05
609	a	g	312	2862	K	K	0	0	0.01
611	c	t	314	2864	S	L	1	0	1.00
641	a	g	344	2894	Y	C	1	0	0.04
647	c	t	350	2900	S	L	1	0	1.00
652	c	t	355	2905	P	S	1	0	1.00
666	c	t	369	2919	D	D	0	0	0.01
680	c	t	383	2933	T	I	1	0	1.00
689	c	t	392	2942	T	I	1	0	1.00
745	c	t	448	2998	P	S	1	0	1.00
760	g	a	463	3013	G	R	1	0	1.00
771	a	g	474	3024	A	A	0	1	0.02
774	a	g	477	3027	I	M	0	0	0.03
786	c	t	489	3039	S	S	0	0	0.02
802	g	a	505	3055	E	K	1	0	1.00
806	c	t	509	3059	P	L	1	0	1.00
824	c	t	527	3077	P	L	1	0	1.00
825	a	g	528	3078	P	P	0	1	0.02
875	t	c	578	3128	L	S	1	0	1.00
949	g	a	652	3202	D	N	1	0	1.00
951	c	t	654	3204	D	D	0	0	0.01
973	c	t	676	3226	P	S	1	0	1.00
