# Supplementary material for: Within-patient mutation frequencies reveal fitness costs of CpG dinucleotides and drastic amino acid changes in HIV
Source: PLoS Genet. 2018 Jun 28;14(6):e1007420. doi: 10.1371/journal.pgen.1007420 (PMC6023119; doi:10.1371/journal.pgen.1007420)
Supplement: S3 Table — Parameters for the gamma distribution of fitness costs for pol mutations based on mutation frequencies the Bacheler, Zanini and Lehman datasets, reflecting scale (κ) and shape (θ). The “fraction lethal” is the fraction of the mutations that had a mean frequency smaller than or equal to the mutation rate, so that they are estimated to be lethal. Sites are resampled with replacement and gamma distributions are fit 1000 times to create 95% confidence intervals via bootstrapping (shown in parentheses). (PDF) [file pgen.1007420.s010.pdf]

	Sites	Scale	Shape	Scale	Shape	Lethal
Bacheler	870	0.334	0.275	0.327	0.333	0.082
		(0.257, 0.411)	(0.265, 0.289)	(0.267, 0.388)	(0.321, 0.348)	(0.066, 0.099)
Zanini	758	0.041	0.645	0.114	0.571	0
		(0.037, 0.045)	(0.605, 0.687)	(0.098, 0.129)	(0.535, 0.61)	(0, 0)
Lehman	415	0.172	0.273	0.245	0.301	0.029
		(0.107, 0.249)	(0.25, 0.305)	(0.182, 0.317)	(0.278, 0.33)	(0.014, 0.046)
