# Supplementary material for: Within-patient mutation frequencies reveal fitness costs of CpG dinucleotides and drastic amino acid changes in HIV
Source: PLoS Genet. 2018 Jun 28;14(6):e1007420. doi: 10.1371/journal.pgen.1007420 (PMC6023119; doi:10.1371/journal.pgen.1007420)
Supplement: S4 Table — (PDF) [file pgen.1007420.s011.pdf]

	Nucleotide substitution	Abram estimate	Zanini estimate
1	A→T	7.93E-07	7.00E-07
2	A→C	5.29E-07	9.00E-07
3	A→G	1.11E-05	6.00E-06
4	C→T	2.41E-05	1.20E-05
5	C→A	6.46E-06	5.00E-06
6	C→G	1.70E-07	5.00E-07
7	G→T	8.46E-07	2.00E-06
8	G→C	8.46E-07	1.00E-07
9	G→A	5.48E-05	1.60E-05
10	T→C	1.11E-05	1.00E-05
11	T→A	1.34E-05	3.00E-06
12	T→G	3.60E-06	3.00E-06
